# Demonstration of photon Bloch oscillations and Wannier-Stark ladders in dual-periodical multilayer structures based on porous silicon

**DOI:** 10.1186/1556-276X-7-413

**Published:** 2012-07-23

**Authors:** J Octavio Estevez, Jesús Arriaga, Antonio Mendez-Blas, Edgar Reyes-Ayona, José Escorcia, Vivechana Agarwal

**Affiliations:** 1 Instituto de Física, Universidad Autónoma de Puebla, Puebla, A.P. J-48, México; 2 Centro de Investigación en Ingeniería y Ciencias Aplicadas, UAEM, Av. Universidad 1001, CuernavacaMéxico, Morelos 62210, Col. Chamilpa

**Keywords:** Photon Bloch oscillations, Wannier-Stark ladders, Dual-periodical multilayers, Porous silicon, Photonic crystals

## Abstract

Theoretical demonstration and experimental evidence of photon Bloch oscillations and Wannier-Stark ladders (WSLs) in dual-periodical (DP) multilayers, based on porous silicon, are presented. An introduction of the linear gradient in refractive indices in DP structure, which is composed by stacking two different periodic substructures *N* times, resulted in the appearance of WSLs. Theoretical time-resolved reflection spectrum shows the photon Bloch oscillations with a period of 130 fs. Depending on the values of the structural parameters, one can observe the WSLs in the near infrared or visible region which may allow the generation of terahertz radiation with a potential applications in several fields like imaging.

## Background

The analogs between electron transport and propagation of the optical waves in dielectric structures opened the possibility of the implementation of Bloch oscillations for electromagnetic waves in photonic crystals (PC) [1,2]. The photonic analog of the above-mentioned effect appears when a PC is subjected to a slowly varying refractive index or a geometric parameter modulation, resulting in a linear tilting of the band structure. Such ‘chirped’ PCs give rise to a set of equidistant frequency levels [3], i.e., the optical counterpart of the Wannier-Stark ladders (WSLs) in semiconductor superlattices. Recently, different methods have been adopted to tilt the photonic band for the observation of WSLs and photon Bloch oscillations (PBOs) [4-7]. In confined Bragg mirrors, the band structure modifications are due to the gradual change of the lateral confinement [4]. On the other hand, in geometrically chirped PCs, the band structure modulation arises from a gradual increase in the thicknesses of the layers [5,6]. Furthermore, in graded-index optical superlattices, the index gradient comes from a linear modification in the refractive indices of the layers [7,8], and so on. The use of above-mentioned PCs with gradient in optical thickness has evoked special interests in many applications due to their novel properties [9]. Such multilayer structures originate a new type of Fabry-Perot cavity where the reflectors are replaced by nonpropagating regions associated with the local periodicity of the structure. If the linear gradient is considered, the distance between the band edges where the PBOs occur can be maintained constant. Hence, the period of PBOs remains constant with the change in the frequency of the incident wave. In case of nonlinear gradient, the distance between the band edges and, therefore, the period of the resulting PBOs can be tuned by changing the frequency of the incident light. For example in 2005 Lousse and Fan [8] reported the tunable terahertz Bloch oscillations in the chirped photonic crystals, with the potential applications in several fields, like biomedical sensing. Such useful photonic structures can be fabricated with different materials. Recently, one-dimensional photonic superlattices made of porous silicon (PSi) have allowed the demonstration of optical analogs [10] of electronic phenomena [11,12], such as PBOs, Zener tunneling, and Anderson localization [6,7,13,14].

Porous silicon provides good flexibility in the design of optical devices due to its easy fabrication technique [15-17] and tunable optical properties. PSi can be obtained by electrochemical etching of doped silicon wafers, which allows the fabrication of several types of one-dimensional (1D) porous silicon photonic bandgap structures, such as distributed Bragg reflectors [18], omnidirectional mirrors [19-22], Fabry-Perot optical microcavities [23,24], waveguides [25], rugate filters [26], and optical biosensors [27-31].

**Figure 1:**
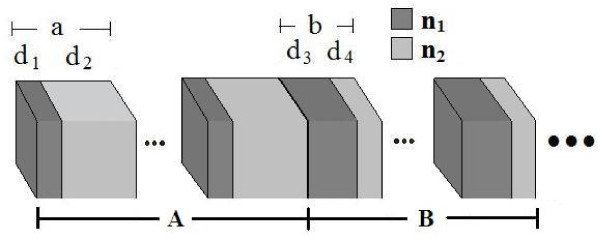
**Dual-periodical multilayer structure.** Schematic view of the 1D dual-periodic structure showing the layer parameters, where *n* and *m* represent the period numbers of *a* and *b* in *A* and *B* substructures, respectively; *n*_1_ and *n*_2_ are high and low refractive indexes of alternating dielectric layers in *a* and *b*; the layer thickness is *d*_1_ and *d*_2_ for *a*, and *d*_3_ and *d*_4_ for *b*, respectively.

In the present work, we demonstrate the theoretical and experimental evidence of WSLs, using dual-periodical multilayer structures (shown schematically in Figure 1), with a linear gradient in refractive indices, based on porous silicon. Theoretical evidence of the presence of PBOs in such structures is also presented.

### Dual-periodic structures

The optical properties of dual-periodical (DP) structures have been theoretically and experimentally reported by several groups [32,33]. Recently, Pérez et al. [34] reported DP structures from PSi multilayers. Dual-periodic structure (Figure 1) is composed of two substructures, *A* and *B*, repeating alternatively in the sequence $A_{n}B_{m}A_{n}B_{m}\mathrm{...}A_{n}B_{m}={\left(A_{n}B_{m}\right)}^{N}$. The *A*_*n*_ and *B*_*m*_are in turn composed of two different periodic units, *a* and *b*, respectively, where subscripts, *n* and *m*, are the number of periods for *a* and *b* in the *A* and *B* substructures, respectively. Both *a* and *b* consist of a pair of layers with high and low refractive indices. The thickness of the double layer *a* is *d*_*a*_ = *d*_1_ + *d*_2_*d*_1_ and *d*_2_ being the thicknesses for the layers with the high (*n*_1_) and low (*n*_2_) refractive indices, respectively. Similarly, the double layer *b* has thickness *d*_*b*_ = *d*_3_ + *d*_4_; *d*_3_ and *d*_4_ being the thicknesses for layers with the high and low refractive index as well. In particular, the following sequence was used: *A*_2_*B*_4_*A*_2_*B*_4_*A*_2_*B*_4_*A*_2_*B*_4_*A*_2_*B*_4_*A*_2_*B*_4_= ${\left(A_{2}B_{4}\right)}^{6}$ for the infrared region. If the substructure *B* is considered as a defective layer, the frequency intervals where the resonances of the transmission peaks appear can be reduced by increasing the number of periods *a* in the substructure *A*. On the other hand, if substructure *A* is a defective layer, the frequency intervals of the resonances can be increased by reducing the number of periods *b* in the substructure *B*. When identical *A*_*n*_*B*_*m*_structures are coupled, a degenerate mode repulsion arises. Each degenerate optical resonance splits up and a miniphotonic band forms [32-34]. Due to the periodicity of the structure, the miniphotonic bands are separated by photonic band gaps in which propagation is prohibited. Moreover, when *N*>1, there are *N*−1 defect layers; therefore, *N*−1 resonance modes and *N*−1 transmission peaks will appear in the spectra. By adjusting the structural parameters, it is possible to tune the number, frequency, and full width at half-maximum (FWHM) of the resonance modes, opening the possibility to fabricate optical filters based on porous silicon multilayers. Such DP photonic structures are very promising in the field of optoelectronics, optical communications, and optical biosensors [34].

Furthermore, 1D translational symmetry of the system should be broken by introducing a small gradient in the refractive indices along the depth of the DP structure to obtain PBOs in periodic 1D photonic crystals. The gradient in the refractive indices results in a spatial tilting of the miniband and photonic band gaps in which the resonances, due to defects in DP structure, change slightly while preserving the mode coupling. In this way, the extended photonic states are turned into a discrete sequence of energy levels with level spacing △E, which is an optical equivalent of a WSL in frequency domain. The refractive index gradient in layers is given by △*n* = ($n_{z_{m}}$ - $n_{z_{1}}$)/$n_{z_{1}}$, where the subscripts *z*_1_and *z*_*m*_ are the first and the m-th layer along the depth within the sample. This gradient is the optical counterpart of the external electric field used in electronic superlattices.

## Methods

The structures were fabricated by electrochemical etching of boron-doped silicon wafers with resistivity from 0.007 to 0.013 *Ω*· cm and (100) orientation. The substrates were etched in an electrolyte consisting of HF (40%) and ethanol (99.98%) in the volumetric ratio of 1:1. To estimate the refractive index corresponding to a given current density, the effective medium Bruggeman’s model is used. To measure the porosity of the layers, gravimetrical method was employed. The reflectivity spectra were measured using p-polarized transverse-magnetic (TM) modes light at an incidence angle of 20° using VARIAN-CARY 5000 spectrometer (Varian Inc., NC, USA ). Two different sets of structural parameters were used to fabricate the PSi structures to observe WSLs and PBOs in the near infrared region. The experimental reflectivity results were compared with the theoretical simulations. To obtain ${\left(A_{2}B_{4}\right)}^{6}$ structure with resonance transmission modes in the near infrared region *d*_1_ = 105, *d*_2_ = 315, *d*_3_ = 216, and *d*_4_ = 103 nm were considered. Their refractive indices were taken as *n*_1_= 2.2 and *n*_2_ = 1.4, which correspond to the porosities of 48% and 76%, and are obtained with current densities (*J*) of 29 and 134 mA/*cm*^2^, respectively. For obtaining a linear refractive index gradient of 16% in ${\left(A_{2}B_{4}\right)}^{6}$ structure (named as G(*A*_2_*B*_4_)^6^), *n*_1_ changes from 2.2 to 2.55 (37% porosity, *J* = 5.2 mA/*cm*^2^) and *n*_2_ from 1.4 to 1.57 (69% porosity, *J* = 89.0 mA/*cm*^2^).

### Theoretical model

#### Light propagation in dual-periodical multilayers

Photon propagation in one-dimensional structures has been modeled by simple transfer matrix method considering p-polarized light [35]. If we consider an electromagnetic (EM) wave propagating in the structure with propagation constant **k**=**k**_∥_ + *k*_*z*_**ẑ**, there are two independent EM modes: TM and transverse electric (TE). The electric and magnetic field for the TE and TM mode are perpendicular to the plane defined by the wave vector and the direction of periodicity. Using the transfer matrix method, we can relate the amplitudes of the fields $E_{\mathrm{j\mu}}^{+}$ and $E_{\mathrm{j\mu}}^{-}$ in the j-th cell of the system to the amplitudes of the field in the (j + 1)-th cell according to [36],

(1) $$\left(\begin{array}{l} E_{\mathrm{j\mu}}^{+}\\ E_{\mathrm{j\mu}}^{-} \end{array}\right)=M_{\mu }\left(\begin{array}{l} E_{j+1\mu }^{+}\\ E_{j+1\mu }^{-} \end{array}\right)$$

where $E_{\mathrm{j\mu}}^{+}$ and $E_{\mathrm{j\mu}}^{-}$ is the amplitude of the wave in layer *j* with polarization *μ*= *sp*traveling to the right and to the left, respectively. For the case considered in this work, the total transfer matrix of the system can be written as a product of matrices of the type [36]:

(2) $$M_{\mathrm{j\mu}}=\left(\begin{array}{ll} \cos(\phi_{j}) & -i\sin(\phi_{j})/q_{\mathrm{j\mu}}\\ -iq_{\mathrm{j\mu}}\sin(\phi_{j}) & \cos(\phi_{j}) \end{array}\right)$$

where *q*_*jμ*_ = $k_{j}^{2}/k_{\mathrm{jz}}$ for p-polarization and *q*_*jμ*_ = *k*_*jz*_ for *s*-polarization; *ϕ*_*j*_=*k*_*jz*_*d*_*j*_*k*_*j*_=(*ω*/*c*)*n*_*j*_*k*_*jz*_ being the component of the wave vector along the growth direction of the system in the j-th layer given by $k_{\mathrm{jz}}=\sqrt{k_{j}^{2}-k_{∥}^{2}}$; and $n_{j}=\sqrt{\varepsilon_{j}}$, its complex refractive index. A real approach over the transfer matrix was implemented by considering the absorption and dispersion dependence. Reflectivity calculations of the system are given in terms of the matrix elements of the total transfer matrix ℳ according to $R={\left|{ℳ}_{21}/{ℳ}_{11}\right|}^{2}$. Using transfer matrix calculations, it is possible to compute the electric field distribution inside the structure. The electric field in the structure along the *z* direction for a certain wavelength can be expressed as follows:

(3) $$E(z,\omega )=\{1+r(\omega )\}{ℳ}_{11}^{k}+\gamma_{0}\{1-r(\omega )\}{ℳ}_{12}^{k}$$

where ${ℳ}_{\alpha \beta }^{k}$ are the elements of the transmission matrix from the first to the k-th interface. As can be seen from this expression, the reflectivity coefficient of the whole structure must be calculated previously before evaluating the field amplitude inside the structure. Hence, the intensity is simply the square of the electric field, |*E*|^2^ which is normalized to the amplitude of the incident signal. The amplitude of the time-resolved reflection can be calculated using the following expression:

(4) $$r(t)=\frac{1}{2\Pi }\overset{+\infty }{\underset{-\infty }{\int }}g(\omega )r(\omega )e^{-\mathrm{i\omega t}}\mathrm{d\omega}$$

where *r*(*ω*) is the reflectivity coefficient, and *g*(*ω*) is the incident pulse with a Gaussian spectral function in frequency domain:

(5) $$g(\omega )=\frac{\hbar }{\sqrt{\Pi }\delta }\exp\left[-{\left(\frac{\hbar \omega -E_{0}}{\delta }\right)}^{2}\right]$$

where *E*_0_ is the central photon energy of the pulse and *δ* is the pulse width. The parameter *δ*controls the pulse duration, which should be sized to a value close to the Bloch oscillations periods. The expression in Equation 4 is valid only for times longer than the duration of the incident pulse [6].

#### Porous silicon as an effective medium

As PSi layer consists of two distinct components (air and silicon); its complex dielectric function (*ε*_PSi_) has an intermediate value between silicon (*ε*_Si_) and air (*ε*_air_), i.e., a volume fraction *f*_p_ of silicon and a volume fraction (1 - *f*_p_) of pores (where refractive index of air = 1). Bruggeman effective medium approximation was used to estimate the effective optical parameters of PSi [37,38]:

(6) $$f_{P}\left(\frac{\varepsilon_{\mathrm{Si}}-\varepsilon_{\mathrm{PSi}}}{\varepsilon_{\mathrm{Si}}+2\varepsilon_{\mathrm{PSi}}}\right)+(1-f_{P})\left(\frac{1-\varepsilon_{\mathrm{PSi}}}{1+2\varepsilon_{\mathrm{PSi}}}\right)=0$$

From this equation we obtain *ε*_PSi_in terms of *ε*_Si_ and the porosity *f*_P_:

(7) $$\varepsilon_{\mathrm{PSi}}=\frac{1}{4}\begin{array}{l} \left\{(2-\varepsilon_{\mathrm{Si}})+3f_{P}(\varepsilon_{\mathrm{Si}}-1\right) \end{array}$$

(8) $$+{\left[{\left((2-\varepsilon_{\mathrm{Si}})+3f_{P}(\varepsilon_{\mathrm{Si}}-1)\right)}^{2}+8\varepsilon_{\mathrm{Si}}\right]}^{1/2}\}.$$

The refractive index *n*(*λ*) and the extinction coefficient *k*(*λ*) can be obtained by the Cauchy model, which is useful for dielectric materials (with exponential absorption), far from the absorption bands [39].

## Results and discussion

**Figure 2:**
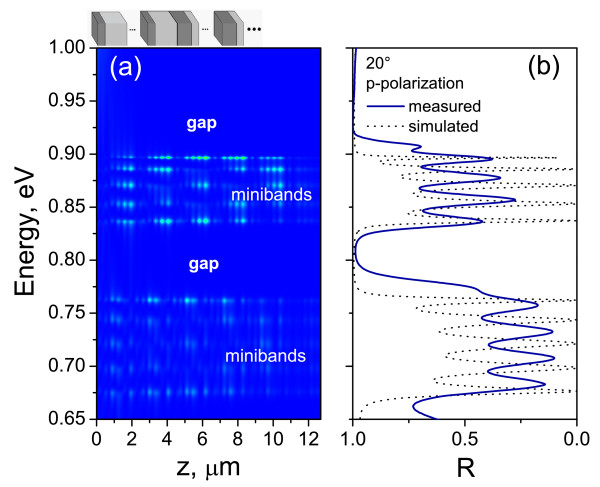
**Scattering states map and reflectance spectra of (A**_**2**_**B**_**4**_**)**^**6**^**structure.** (**a**) Calculated electric field intensity distribution of TM modes in (*A*_2_*B*_4_)^6^ structure with no refractive index gradient i.e., △*n*= 0 (flat miniband situation). A change of color from dark to bright corresponds to the increase in the light intensity. (**b**) Measured and simulated reflectance spectra of the structure. The schematic illustration of the corresponding DP structure is shown just above the scattering states map.

Figure 2a shows the square of the electric field inside the DP ${\left(A_{2}B_{4}\right)}^{6}$ structure, without any refractive index gradient (△*n* = 0), calculated by transfer matrix methods using Equation 3 for infrared region. The scattering states map provides the electric field intensity at each position inside the structure when plane electromagnetic waves of unit amplitude impinge onto the structure to 20° of incidence. A change from dark to bright corresponds to the increase in the light intensity. Although, the input intensity has been normalized to unity, in some of the regions, the intensity inside the structures can be more than one due to internal resonances. No field localization is observed among the different zones in the DP structure. Composed by some delocalized states in space, each bright band is an optical analog of electronic energy band caused by the periodic potential in electronic superlattices. In Figure 2b the optical reflectivity measurements performed on DP structure, fabricated for infrared region of the electromagnetic spectrum, are presented. The dotted lines in Figure 2b correspond to the results obtained by numerical simulations. The measured reflectance spectrum shows the presence of five resonance peaks at photons energy 0.837, 0.853, 0.870, 0.885, and 0.896 eV, with FWHM of 6, 8.2, 8, 7.1, and 4 meV, respectively. The FWHMs of the resonance peaks can be tuned by increasing or decreasing the number of layers in either of the substructures.

The decreasing or the increasing number of layers of the substructure, acting as a defect in the system (substructure *B* for the samples considered here), results in the widening or narrowing of the peaks and consequently, the value of FWHM [34].

**Figure 3:**
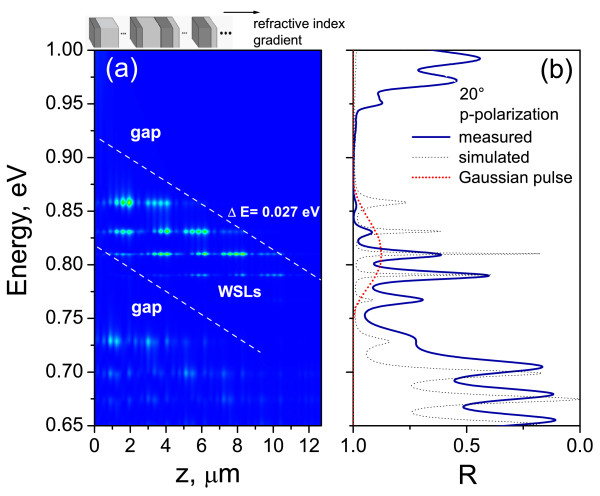
**Scattering states map and reflectance spectra of G(A**_**2**_**B**_**4**_)^**6**^**structure.** (**a**) Calculated electric field intensity distribution of TM modes in G(*A*_2_*B*_4_)^6^ structure with a linear gradient in refractive index (△*n* = 16%). Inclined white lines are shown as indicators of the miniphotonic band inclination. One can observe the formation of the photonic WSLs confined between two minigaps. (**b**) Measured and simulated reflectance spectra of the structure and the input Gaussian pulse (red dotted line) in frequency domain.

Figure 3a shows the square of the electric field inside the G${\left(A_{2}B_{4}\right)}^{6}$ structure with △*n* = 16% in the same range of frequencies as in Figure 2. One can clearly observe the light confinement due to the inclination of the minibands and photonic bandgaps (PBGs) (Figure 3a). The occurrence of WSL as a series of narrow resonant peaks is clearly observed in the reflectivity spectra of the structure (Figure 3b). When the incident pulse enters in G${\left(A_{2}B_{4}\right)}^{6}$ structure in the frequency region between two local gaps, most of the light is reflected back on the lower band gap, and only a small percentage which crosses the gap elastically (Zener tunneling) feeds the horizontal resonances associated with the WSLs. Hence, the confined light starts to oscillate within the inclined allowed miniband with a regular period of *τ*_*B*_ = *h*/△E. These are PBOs which represent the time domain counterpart of the photonic WSLs. Time domain oscillations can be detected by measuring the time-resolved reflection of the sample. The center of the Wannier-Stark resonance defines, in space, the center of the PBO. The distance between successive frequency levels can be obtained by the following Fabry-Perot formula: △E = *hc*/$2\bar{n}d$, where *d* is the local distance between the gap edges at the given input frequency and $\bar{n}$ is the average refractive index. The measured values of △E, from Figure 3b, are found to be in agreement with the theoretical simulations presented in Figure 3a (as a function of film depth). The structural parameters are modulated to obtain the energy spacing △E ≈ 27 meV, and therefore, *τ*_*B*_ = 148 fs.

**Figure 4:**
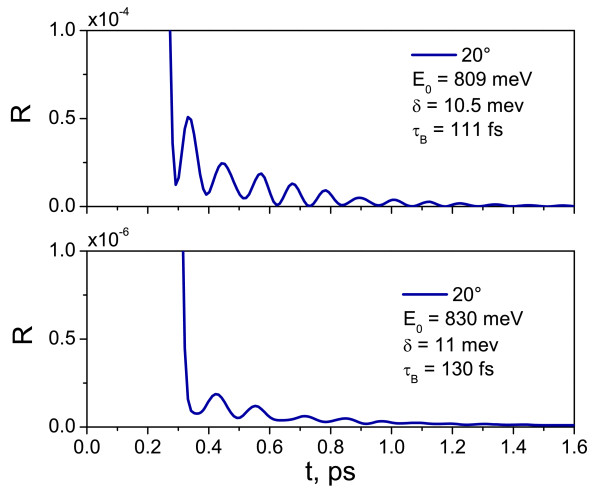
**Time-resolved reflection spectra in G(A**_**2**_**B**_**4**_**)**^**6**^**structure.** Calculated time-resolved reflection spectra of the G(*A*_2_*B*_4_)^6^ structure for incident pulse energy of (top image) *E*_0_= 809 meV (bottom image) *E*_0_= 830 meV.

Figure 4 shows the theoretical time-resolved reflection of the G${\left(A_{2}B_{4}\right)}^{6}$ structure calculated using Equation 4 for a Gaussian pulse with *E*_0_= 809 meV (*δ* = 10.5 meV), as shown in Figure 3b, and *E*_0_= 830 meV (*δ*= 11 meV). One can observe the PBOs with an oscillation period of 111 and 130 fs(Figure 4), compared to 148 fs (△E = 27 meV) measured from the reflectance spectrum of the corresponding structure (Figure 3b). These oscillations are observable due to the tunneling of photons through the lower inclined gap. The form and the energy of the pulse are found to slightly influence the period of the PBOs, e.g., an increase in the pulse energy by 21 meV results in a increase of PBO period by 19 fs.

## Conclusions

Successful demonstration of WSLs and consequently the PBOs in one-dimensional dual-periodical PSi structures for near infrared range is presented. The possibility of observing the WSLs in reflectance spectrum is evaluated by selecting the adequate parameters with a linear gradient in refractive indices. Such photonic structures can be very promising in the observation of Bloch oscillations in the different regions of the electromagnetic spectrum.

## Competing interests

The authors declare that they have no competing interests.

## Authors’ contributions

JOE prepared the PSi multilayers. JA, ER-A, and JE performed the treatment of experimental data and simulations. AMB and VA proposed the experimental conditions and measured the structures. JOE, JA,and VA prepared the manuscript initially and participated in its design and coordination. All authors read and approved the final manuscript.
